# Information Seeking Behavior of Latino Family Caregivers of People With Alzheimer’s Disease and Related Dementias

**DOI:** 10.1093/geroni/igaa057.876

**Published:** 2020-12-16

**Authors:** Magaly Ramirez, Sofia De Anda, Haomiao Jin, Joseph Herrera, Shinyi Wu

**Affiliations:** 1 University of Washington, Seattle, Washington, United States; 2 University of Southern California, Los Angeles, California, United States; 3 Rancho Los Amigos, Downey, California, United States

## Abstract

Latinos are disproportionately affected by Alzheimer’s disease and related dementias (ADRD) compared to non-Latino Whites. Family caregivers need access to high-quality information, education, and support. The study objective was to understand the information seeking behavior of Latino caregivers of individuals with ADRD. We conducted qualitative interviews in Los Angeles County with Latino caregivers of individuals with ADRD (N=21) and with healthcare and community stakeholders serving Latinos with ADRD and their caregivers (N=6). Caregivers sought information about ADRD, healthcare services and treatment, available community resources, caregiving skills, and short- and long-term care. Having limited time to seek information, lacking skills to navigate the Internet, and insufficient availability of information created barriers to accessing information. With access to information, caregivers increased their knowledge about ADRD, enrolled the person with ADRD in a memory/dementia clinic, participated in treatment decisions, attended community events, learned how to respond to challenging behaviors, and enrolled in daycare/respite programs. Caregivers experienced barriers to using information due to the insurance and immigration status of the person with ADRD and due to services only being available in English. Our study contributes new knowledge on the accessibility and use of information by Latino caregivers and their unique barriers in transforming information access into meaningful results. Healthcare and community-based providers can use our study findings to tailor informational interventions to the needs and preferences of Latinos and to the unique barriers they face in using information to support their caregiving role.

